# Tauroursodeoxycholic Acid Reduces Neuroinflammation but Does Not Support Long Term Functional Recovery of Rats with Spinal Cord Injury

**DOI:** 10.3390/biomedicines10071501

**Published:** 2022-06-25

**Authors:** Siyu Wu, Concepción García-Rama, Lorenzo Romero-Ramírez, Johannes P. J. M. de Munter, Erik Ch. Wolters, Boris W. Kramer, Jörg Mey

**Affiliations:** 1 Hospital Nacional de Parapléjicos, 45071 Toledo, Spain; wusy1029@gmail.com (S.W.); cgrama@externas.sescam.jccm.es (C.G.-R.); lromeroramirez@sescam.jccm.es (L.R.-R.); 2 School of Mental Health and Neuroscience and EURON Graduate School of Neuroscience, Maastricht University, 6229 ER Maastricht, The Netherlands; b.kramer@maastrichtuniversity.nl; 3 Neuroplast BV, 6167 RD Geleen, The Netherlands; h.demunter@neuroplast.com (J.P.J.M.d.M.); e.wolters@neuroplast.com (E.C.W.)

**Keywords:** bile acid, spinal cord injury, bone marrow-derived stromal cells, rat, neuroinflammation

## Abstract

The bile acid tauroursodeoxycholic acid (TUDCA) reduces cell death under oxidative stress and inflammation. Implants of bone marrow-derived stromal cells (bmSC) are currently under investigation in clinical trials of spinal cord injury (SCI). Since cell death of injected bmSC limits the efficacy of this treatment, the cytoprotective effect of TUDCA may enhance its benefit. We therefore studied the therapeutic effect of TUDCA and its use as a combinatorial treatment with human bmSC in a rat model of SCI. A spinal cord contusion injury was induced at thoracic level T9. Treatment consisted of i.p. injections of TUDCA alone or in combination with one injection of human bmSC into the *cisterna magna.* The recovery of motor functions was assessed during a surveillance period of six weeks. Biochemical and histological analysis of spinal cord tissue confirmed the anti-inflammatory activity of TUDCA. Treatment improved the recovery of autonomic bladder control and had a positive effect on motor functions in the subacute phase, however, benefits were only transient, such that no significant differences between vehicle and TUDCA-treated animals were observed 1–6 weeks after the lesion. Combinatorial treatment with TUDCA and bmSC failed to have an additional effect compared to treatment with bmSC only. Our data do not support the use of TUDCA as a treatment of SCI.

## 1. Introduction

Lesions of the spinal cord due to traumatic injury, tumors, or vascular ischemia frequently cause paralysis and the loss of autonomic functions. No disease modifying therapy for this pathology is currently available. The severe consequences of spinal cord injury (SCI) are in a large part due to a secondary inflammatory reaction, which is borne by local microglia cells, macrophages, and lymphocytes that infiltrate the lesion area. This reaction, in conjunction with increasing vascular permeability, causes cell death of neurons and glia cells [1]. For this reason, anti-inflammatory treatment is a therapeutic strategy for SCI and has been used in the clinic [2]. Unfortunately, the approved treatment option, methylprednisolone, is often ineffective and causes severe side effects such as a higher incidence of sepsis, gastrointestinal hemorrhage, or pulmonary embolism [3]. Therefore, new therapies for SCI are needed, and one of the most promising lines of research with this aim consists of the application of stem cells [4,5,6]. We have recently tested the safety and therapeutic benefits of human bone marrow-derived stromal cells (bmSC), which are prepared solely by negative selection without expansion in vitro (Neuroplast BV, patent WO2015/059300A1).

In rat models of SCI, intrathecal infusion of these cells reduced chronic inflammation and neural degeneration and provided a benefit on the functional level [7,8]. However, as in most other studies [5], the beneficial effect was limited. In comparison with control treatments, the recovery of sensory-motor function improved by 1.5 points (BBB scale) [9], and even at nine weeks after SCI, most rats did not recover beyond BBB 9-11 [8]. One reason for the limited effect of bmSC is that implanted cells die or are actively eliminated in the acute phase. We reasoned that a combinatorial therapy of bmSC implantation with additional cytoprotective measures would be advantageous to support the integration of implanted bmSC.

A recent strategy of cytoprotection for neuropathologies is based on bile acids, which have long been used in traditional Chinese medicine [10,11]. These are amphipathic molecules synthesized from cholesterol. Their biological effects are mediated via the Takeda G protein-coupled receptor-5 (TGR5) [12,13] as well as nuclear receptors farnesol X receptor, pregnane receptor, and liver X receptors [14,15]. While bile acids play important roles in lipid metabolism [16], they also have anti-inflammatory and cytoprotective effects by suppressing NFκB signaling, which make them interesting candidates for the treatment of neuropathologies [17,18]. One particular bile acid, tauroursodeoxycholic acid (TUDCA), has been tested in animal models for Parkinson’s disease [19], multiple sclerosis [20], and a clinical trial of amyotrophic lateral sclerosis [21]. In rodent models of SCI, TUDCA reduced cellular apoptosis [22,23,24,25,26]. With rats, improvements on the functional level were observed within the first 5 days after injury, i.e., in the subacute phase, but it is not clear whether these treatments have a lasting effect after SCI.

Our objectives in the present study were (1) to assess the effects of TUDCA on neuro-inflammation and astrogliosis after SCI, (2) to clarify the long term therapeutic effects of TUDCA treatment on the recovery of sensory-motor function, and (3) to test whether a combinatorial therapy of TUDCA and bmSC transplantation provides additional benefit.

## 2. Materials and Methods

### 2.1. Experimental Animals and Study Design

**Figure 1:**
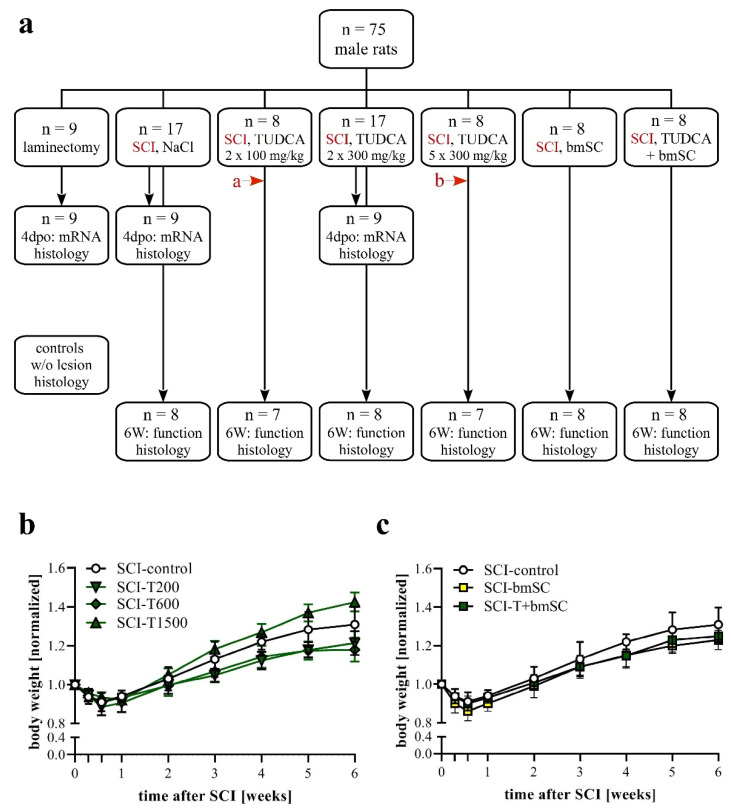
Experimental plan. (**a**) Animals were pseudo-randomly assigned to the different treatment groups. Functional analysis was carried out during a recovery period of six weeks after SCI with the following treatment conditions: (1) two injections of saline at the time of surgery (t0) and 24 h later, (2) two injections of 100 mg/kg TUDCA at t0, 24 h; (3) two injections of 300 mg/kg TUDCA at t0, 24 h; (4) five injections of 300 mg/kg TUDCA at t0, 24 h, 2 dpo, 4 dpo and 6 dpo; (5) one injection of bmSC at t0 + 2 h; (6) combinatorial treatment with bmSC and two injections of 100 mg/kg TUDCA. In addition, three treatment groups (laminectomy only, SCI-control, and SCI with 2 × 300 mg/kg TUDCA) were evaluated at 4 dpo with biochemistry and IF. In histology at 6 W, spinal cord tissue was also compared to tissue from non-lesioned rats. Two animals had to be excluded from the study because open field evaluation at 1 dpo suggested an incomplete lesion (red arrows a: BBB = 7, b: BBB = 8). (**b**,**c**) Changes in body weight following SCI: Animals in all treatment groups suffered from weight loss during the first 4 dpo and subsequently recovered [means +/− SD; see text for statistical evaluation].

Experimental protocols, surgical procedures, and post-operational care were reviewed by the ethics committee for animal care of the Hospital Nacional de Parapléjicos (163CEEA/2017) and approved by the Consejería de Agricultura y Ganadería de Castilla-la Mancha (ref. 210498, following EU directive 2010/63/EU). We used male Wistar rats (*Rattus norwegicus*), six to eight weeks of age, which had been bred in the animal facility of the hospital. Until the day of surgery, animals were kept in pairs and, subsequently, in individual cages. Standard housing conditions consisted of a 12 h light/dark cycle, 40–60% humidity, temperature of 22 °C with ad libitum access to food and water. A total of 75 animals entered the study (Figure 1a; sample size calculation for BBB scores at 6 weeks based on α = 0.05, β = 0.2, d = 3, SD = 2, attrition 10%). In addition, the spinal cords of non-injured rats were processed for comparison of histological results. Since rats had the same sex, and a similar age and body weight, no randomization was performed to allocate them in the study. The order of treatment was mixed to ensure that all treatment groups were served throughout the entire period when surgery was performed. Rats were excluded when the SCI was considered invalid. Since the primary outcome of the study was sensory-motor function, the exclusion criteria were a BBB score above 2 at 24 h after SCI or a force/time plot of the impactor device that indicated that bone was hit.

### 2.2. Surgical Procedures and Postoperative Treatment

Spinal cord contusion injury and injections of bmSC were performed as described previously [8]. In short, anesthesia consisted of 2.5% isoflurane/97.5% oxygen at 0.5 L/min for SCI. For the injections of bmSC, we used one i.p. injection of ketamine 50 mg/kg combined with xylacine 5 mg/kg. Fifteen minutes before surgery, rats received one s.c. injection of buprenorfine 0.05 mg/kg to reduce pain. Corneal dehydration was prevented with ophthalmic ointment (Lubrithal, Dechra, Barcelona, Spain). With ketamine anesthesia, 0.04 mg/kg of atropin was given. Following laminectomy at thoracic level T9, a spinal cord contusion of 2 N (200 Kdyn, zero dwell time) was inflicted with the Infinite Horizon (IH) spinal cord impactor. We checked the procedure visually (hematoma) and by monitoring the IH displacement/time and force/time plots. To normalize biochemical data at 4 days post operation (dpo), a control group was operated on using the laminectomy procedure without SCI. Immediately after surgery, all animals received 2 × 2.5 mL of isotonic saline s.c. to prevent dehydration and antibiotic treatment with marbofloxacin 5 mg/kg (10 mg/mL s.c. Marbocyl, Alcobendas, Spain). Surgery and behavioral assessments were performed between 09:00 and 14:00.

The transplantation of bmSC was done 2 h after SCI. After the anesthetized animals were positioned in a stereotactic frame, the atlanto-occipital membrane was exposed and penetrated with a pointed scalpel blade. A catheter was then inserted and the cell suspension was slowly infused with a syringe pump (100 µL/3 min) into the cisterna magna. While a rat was being prepared by one researcher, a second person removed one batch of bmSC from storage in liquid nitrogen, thawed and washed the cells with saline, and resuspended them in 110 µL of saline. From this, 10 µL was removed for cytometric counting of cell numbers and determination of cell viability. On average, one injection of 100 µL contained 2.6 million viable cells.

Postoperative care, including analgesic and antibiotic treatment, was done as previously described [8]. The bladders were checked and voided manually every 12 h until the rats were urinating spontaneously. The volume of retained and manually expelled urine per 12 h was recorded. Euthanasia at the end of the study was induced by an i.p. injection of 100 mg/kg of sodium pentobarbital (Dolethal, Madrid, Spain).

### 2.3. Experimental Groups

Animals were assigned to seven experimental groups, six of which received the same SCI but differed in the treatment procedure (Figure 1a). One group (sham) had T9 laminectomy but underwent no contusion injury. Group *SCI-control* received two i.p. injections of saline, the first immediately after SCI (t0) and the second 24 h later (1 dpo). Group *SCI-T200* was treated with two i.p. injections of TUDCA 100 mg/kg body weight at t0 and 1 dpo. Group *SCI-T600* had two i.p. injections of TUDCA 300 mg/kg at t0 and 1 dpo. Group *SCI-T1500* had five injections of TUDCA 300 mg/kg at t0, 1 dpo, 2 dpo, 4 d po, and 6 dpo. Group *SCI-bmSC* received human bmSC implants at t0 + 2 h. Group *SCI-bmSC + T* had bmSC at t0 + 2 h and, in addition, two injections of TUDCA 100 mg/kg at t0 and 1 dpo. During the following six weeks, the investigators who performed the behavioral evaluation were blind with regard to the experimental condition of the individual animals. Nine rats from each of the laminectomy (sham), SCI-control, and SCI-T600 groups were sacrificed at 4 dpo for biochemical and histological analysis. Additional spinal cord sections from non-injured rats were used for comparing histological data.

### 2.4. Preparation of bmSC

Human bmSC for SCI treatment were prepared by negative selection eliminating erythrocytes with Ficoll density gradient centrifugation and, subsequently, B-cells (CD20), T-cells (CD3), monocytes (CD14) and natural killer cells (CD56) using antibody-based cell sorting with magnetic beads under GMP conditions. Cells were not expanded by cultivation (Neuroplast BV, patent WO2015/059300A1, Geleen, The Netherlands). All procedures for the collection of human bone marrow were approved by the ethics committee of Maastricht University Medical Center (METC 13-2-032). The viability and cell type composition of each batch were analyzed with flow cytometry (CD34, CD271, CD90, CD105, CD73). For the present study, bmSC were prepared at the Neuroplast facility in Geleen, NL, cryoprotected with DMSO, frozen in liquid nitrogen, shipped on dry ice to Toledo, Spain, and then stored in liquid nitrogen until use. Cell viability (exclusion of 7-amino-actinomycin D, cytometry) was again determined after thawing, i.e., immediately before application in vivo.

### 2.5. Bile Acid Treatment

Tauroursodeoxycholic acid (Calbiochem CAS 14605-22-2, Millipore, Madrid, Spain) was dissolved at 150 mg/mL in 0.9% saline immediately before the intraperitoneal injection or stored for no longer than 24 h at 4 °C.

### 2.6. Evaluation of Locomotor Functions

This was the primary outcome of the study. Recovery of limb movements was evaluated in the open field using the Basso/Beattie/Bresnahan (BBB) locomotor function scale [9]. This was done before surgery (baseline), at 1 dpo, 2 dpo, 3 dpo, 4 dpo, 7 dpo, and subsequently once per week until six weeks after SCI. At the beginning, we established a criterion of BBB ≤ 2 at 1 dpo for inclusion in the study because a higher score was considered to indicate incomplete SCI. Scoring was performed independently by two investigators who were blind with respect to the treatment of the individual animals. Following assessment, both investigators discussed their evaluation, and in cases where different scores were given, the average of both was recorded.

A second assessment was made using the Rotarod test (Ugo Basile SRL, Gemonio, Italy). In this task, rats are positioned on a slowly rotating rod, which obliges them to use their hind legs in order to keep their balance [27]. In six training sessions of 5 min each, at three, two, and one days before SCI, all rats learned this task at a constant speed of 5 rpm of the rotating rod. During tests, which were administered at 7 dpo and then once per week, the rotation speed was accelerated from 5 rpm to 15 rpm over a period of 5 min. The readout in this assay was the time that the rats were able to stay on the rotating rod before falling off (two repetitions, separated by a break of ≥15 min). At 4 dpo, we confirmed that none of the SCI rats to be included in the study showed weight supported steps. Considering the high variability of this assay, we applied an additional performance assessment using the percentage of animals in each group that were able to maintain their balance for more than 30 s at 6 W after SCI.

### 2.7. Von Frey Test of Mechanical Allodynia/Hyperalgesia

Tactile allodynia/hyperalgesia were evaluated using a dynamic plantar aesthesiometer (von Frey test; Hugo Basile 37550, Gemonio, Italy). For each hind leg, a paw withdrawal threshold (PWT) was determined up to a maximum force of 50 g. This was done five times, with intervals of at least five minutes between tests. The lowest and highest values of these readings were excluded, and then the mean was calculated as PWT. This test was administered five weeks after SCI, when all animals were physically able to respond to the stimulation. On the basis of measurements before the lesion, we considered a PWT of below 20 g as an indication of neurogenic pain.

### 2.8. Quantification of Gene Expression

Animals for biochemical evaluation were sacrificed at 4 dpo with an overdose of sodium pentobarbital. After opening the thoracic cavity, a blood sample of 1 mL was taken from the heart, mixed with 100 µL 0.5 M EDTA to prevent coagulation, spun down, and the supernatant frozen (samples not intended for this study). This was followed by transcardial perfusion with phosphate buffered saline (PBS; 200 mL/rat), preparation of the brains and spinal cords. Spinal cord samples consisted of a 2 cm segment with the lesion site in the center. Tissues were homogenized mechanically with Trizol (Invitrogen, 15596018, Madrid, Spain) and the RNA extracted according to manufacturer’s instructions. To remove genomic DNA, purified RNA was digested with DNase I (ThermoScientific, EN0521, Madrid, Spain). An aliquot corresponding to 0.5 μg of purified RNA was used for first-strand cDNA synthesis using Superscript III reverse transcriptase and oligo (dT) primers in a final volume of 40 µL (Invitrogen Life Technologies, K1632). A real-time quantification of cDNA was performed using a SYBR Green PCR assay. Each 15 µL SYBR green reaction mixture consisted of 1 µL cDNA, 7.5 µL SYBR Green PCR-mix (2×), 0.75 µL forward and reverse primers (10 pM) and 4.75 µL distilled water. PCR was performed with 5 min at 95 °C, followed by 40 cycles of 15 s at 95 °C, 60 s at 60 °C and a separate dissociation step for the melting curve. The specificity of the PCR product was confirmed by ascertaining a single melting peak in the temperature dissociation plots. All samples were run in triplicates and the level of expression of each gene was compared with the expression of acidic ribosomal phosphoprotein P0 (36B4). Amplification, detection of specific gene products and quantitative analysis were performed using an ABI 7500 sequence detection system (Applied Biosystems, Alcobendas, Spain). PCR efficiency was verified by dilution series (1, 1/3, 1/9, 1/27, 1/81, and 1/243) and relative mRNA levels were calculated using the comparative ΔCt method with normalization to 36B4. Gene identifiers, primer sequences, product sizes, and melting temperatures are listed in Table 1.

### 2.9. Tissue Preparation and Histological Staining

At four days or six weeks after SCI, rats were sacrificed with an overdose of sodium pentobarbital followed by transcardial perfusion with PBS and 4% paraformaldehyde/PBS (PFA). The spinal cords were prepared, post-fixed for 1 h, then stored at 4 °C in PFA for 1–3 days. For histological processing, 2 cm long spinal cord segments that included the lesion site in the center were dissected, dehydrated, embedded in paraffin, and cut in 3 μm parasagittal sections using a Leica RM2265 microtome. Sections were mounted on polylysine-coated glass slides (Superfrost Plus, Fisher Scientific, Madrid, Spain) and stored at room temperature (RT). Apoptotic cell nuclei were stained with terminal deoxynucleotidyl transferase dUTP nick end labeling (TUNEL) using the One-step fluorescence TUNEL apoptosis kit (Elabscience, E-CK-A325, Houston TX, USA) according to the manufacturer’s protocol.

### 2.10. Immunofluorescence

For immunofluorescence (IF) staining, sections were rehydrated and incubated for 30 min at 90 °C (water bath) in 10 mM Na citrate/0.05% Tween 20, pH 6.0, for antigen retrieval. Standard procedure included blocking for 30 min at room temperature (RT) with 5% normal goat serum/0.05% Tween 20 in Tris-buffered saline (TBS-T), incubation with primary antibodies for 12 h at 4 °C in a humidified chamber, and 1 h of incubation with fluorescence-labeled secondary antibodies at RT. Nuclei were stained with 10 μg/mL Hoechst-33342 for 15 min at RT. Sections were cover slipped with ImmuMount (Thermoscientific). The following primary antibodies were used in double staining experiments: Polyclonal rabbit anti-GFAP (Sigma G9269; 1/500), polyclonal guinea pig anti-Iba1 (1/500; Synaptic systems 234004, Göttingen, Germany), monoclonal mouse anti CD68 (1/250; Serotec MCA341R1, Alcobendas, Spain). Secondary antibodies were labeled with fluorescent dyes: Goat anti-guinea pig IgG, Alexa-488 (Invitrogen A11073; 1/500), goat anti-rabbit IgG, TRITC (1/500; Sigma T5268, Madrid, Spain), goat anti-mouse IgG, Alexa-594 (Invitrogen A11005; 1/500), goat anti-mouse IgG, Alexa-488 (1/500; Jackson 115-545003, Cambridge, UK).

### 2.11. Microscopy and Image Analysis

Immunohistochemical staining was evaluated using a Leica epifluorescence microscope (20×, 40× objective). Exposure conditions were kept constant for quantitative evaluation with GFAP, CD68, and Iba-1. Photographs were analyzed using Fuji Image-J, applying the same brightness/contrast adjustments and threshold values for each marker.

The intensity of immunoreactivity was measured as *integrated density* in regions of interest (ROI) in the ventral white matter at 8 mm distances anterior and posterior (ROI 0.3 mm^2^) and within the lesion center (ROI 0.075 mm^2^). Following background subtraction, signal intensities were normalized to values found in spinal cord sections from sham-operated rats (4 dpo) or rats without SCI (6 W survival). The number of CD68 positive cells was counted in the same regions. For the evaluation of apoptosis, we counted cell nuclei that were TUNEL positive and expressed the data as percentages of all nuclei in ROI of 1 mm^2^, which were located in ventral white matter at 4 mm anterior and posterior of the lesion and in the lesion center.

A Sholl analysis was performed to quantify morphological changes of microglia cells. Iba-1-stained sections from spinal cord gray matter were photographed at an 8 mm distance from the site of injury (laminectomy or SCI +/− treatment) and visualized with Image-J. From three rats per treatment group, 15 cells were randomly selected and eight concentric rings superimposed over the cell nuclei (Fiji plugin *concentric circles*). The number of intersections of cell processes with each ring was counted manually, excluding extensions from neighboring cells.

### 2.12. Statistical Analysis

Unless stated otherwise in the figure legends, data are presented as mean values ± standard error of the mean (SEM). In box and whiskers plot data are shown with median, first and third quartiles and complete range. Statistical analysis, performed with GraphPad Prism software, consisted of one-factor or two-factor ANOVA, followed by post-hoc Dunnett’s or Sidak’s multiple comparison tests. In graphical data representation, statistical significance is indicated as follows: *, #: *p* < 0.05, **, ##: *p* < 0.01 and ***, ###: *p* < 0.001. Normal distribution of data within groups was assessed with a Kolmogorov-Smirnov test. Performance differences of treatment groups in von Frey and Rotarod tests were assessed using confidence intervals of proportions based on binomial calculation.

## 3. Results

### 3.1. Effects of SCI, TUDCA, bmSC and TUDCA/bmSC Combinatorial Treatment on the General Health Status and Body Weight of the Animals

The general health of the rats was not affected by either treatment with TUDCA alone or in combination with human bmSC, and no adverse effects such as sickness behavior or urinary infections were observed. Five weeks after SCI, two animals, one treated with 5 × 300 mg/mL TUDCA and one in the saline control group, had wounds on their flanks due to biting and were treated topically with antibiotic ointment. During the first four days following SCI surgery, the animals lost 9–11% of their weight, which they recovered with a weight gain of 9.5% in the second week (W1–2), subsequently 8.2% (W2–3), 7.3% (W3–4), 4.9% (W4–5), and 2.2% (W5–6). Changes in body weight over time were significant [two-factor ANOVA; effect of time: F (8, 374) = 370, *p* < 0.001; treatment: F (5, 374) = 37, *p* < 0.001], but post hoc tests showed no significant differences in weight loss or gain between the control group and TUDCA-treated (Figure 1b) or bmSC-treated rats (Figure 1c). Laminectomy without SCI caused an initial weight loss of 4%.

### 3.2. Expression of TGR5 after SCI

In the rat brain, astrocytes and neurons are immunoreactive for the bile acid receptor TGR5 [12]. We confirmed expression in the spinal cord with quantitative RT-PCR and found the level of TGR5 mRNA at 0.6 ± 0.2 ‰ (mean ± SD, n = 5) compared to the ribosomal gene 36B4, which was approximately the same as in the cerebral cortex (0.7 ± 0.2 ‰). It did not change significantly after SCI with or without treatment.

### 3.3. Faster Recovery of Bladder Control with TUDCA Treatment

**Figure 2:**
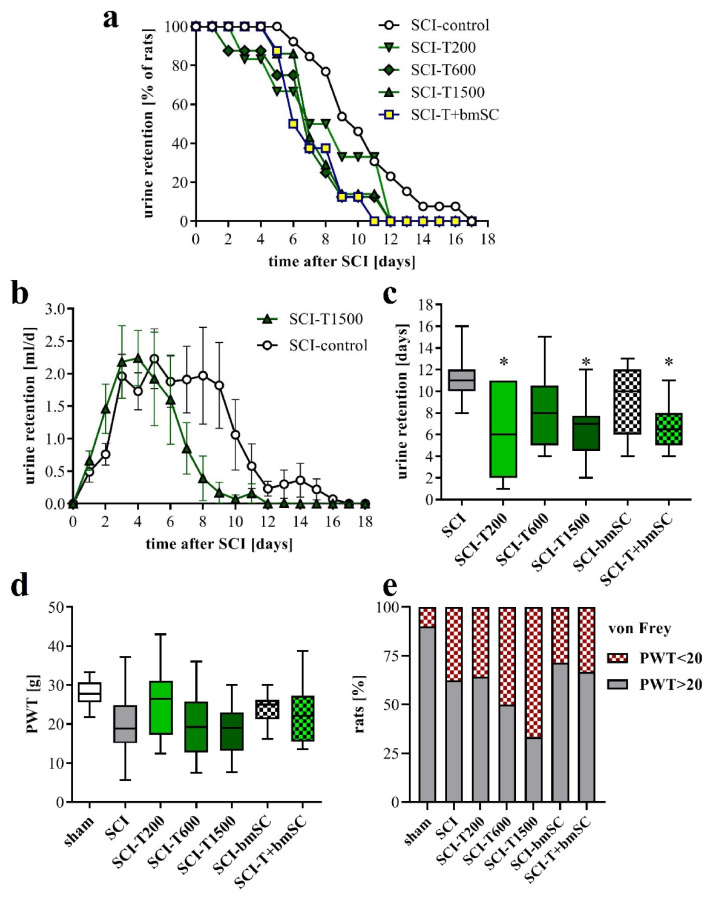
Autonomic functions and neuropathic pain. (**a**) Percentage of rats that required manual bladder voiding each day in the post treatment period. Bladders were voided manually every 12 h. (**b**) Average volume of retained urine per day and rat. Data shown here are for the SCI rats treated with saline and with five injections of TUDCA (mean ± SEM). (**c**) Time after SCI that passed until the animals no longer required manual voiding of the bladder [ANOVA, F (5, 43) = 2.7, *p* < 0.05; post hoc Dunnett’s test vs. SCI control * *p* < 0.05]. (**d**) To assess neuropathic pain we measured the PWT to mechanical stimulation (von Frey test; median ± 25–75 percentile, range; ANOVA n.s.). (**e**) Percentage of rats that had a PWT of below 20 g, which was considered as an indication of allodynia. Experimental groups are abbreviated as follows. SCI-control: two injections of saline; SCI-T200: two injections of 100 mg/kg TUDCA at t0 and 24 h later; SCI-T600: two injections of 300 mg/kg TUDCA at t0, 24 h; SCI-T1500: five injections of 300 mg/kg TUDCA at t0, 24 h, 2 dpo, 4 dpo and 6 dpo; SCI-bmSC: one injection of bone marrow-derived stromal cells at t0 + 2 h; SCI-T + bmSC: combinatorial treatment with bmSC and two injections of 100 mg/kg TUDCA; sham: laminectomy only.

Normal micturition requires coordinated activation of the detrusor muscle of the bladder and relaxation of the external urethral sphincter, which are controlled by spinal and supraspinal centers [28]. Since these connections were affected by the spinal cord contusion, the animals with SCI were unable to urinate spontaneously and needed manual assistance with bladder voiding during the first days after surgery. Laminectomy alone did not cause urinary retention. The volume of manually expelled urine was evaluated to assess the recovery of bladder control (Figure 2). One positive effect of TUDCA treatment, not reported before, was that recovery of this function occurred faster than in rats with saline injection (Figure 2a). The total volume of urine retained during the post-acute period was not significantly different between groups (Figure 2b), but the average duration of compromised bladder control was shorter for animals treated with TUDCA (Figure 2c). The recovery of the areflexive bladder in the group treated with additional bmSC was similar to that of the group treated with TUDCA only. Eventually, however, all rats recovered autonomic control of micturition.

### 3.4. Effect of TUDCA and bmSC Treatment on Allodynia/Hyperalgesia

Spinal cord injury can lead to neuropathic pain. This was assessed at five weeks after SCI by determining the withdrawal threshold (PWT) to mechanical stimulation of the hind paws using an automated von Frey test. Although we found no significant differences between experimental groups (Figure 2d; ANOVA, F (6, 82) = 2.5, *p* < 0.05, put post-hoc Dunnett’s test vs. SCI n.s. for all groups), the number of rats that had a PWT below 20 g, which we considered indicative of allodynia or hyperalgesia, was higher after SCI than after the laminectomy operation. This measure of pain sensitivity appeared to be worse with high doses of TUDCA and lower with treatment of bmSC alone (Figure 2e), but variability and group size do not permit a conclusive interpretation.

### 3.5. Effect of TUDCA and bmSC Treatment on Recovery of Sensory-Motor Functions

**Figure 3:**
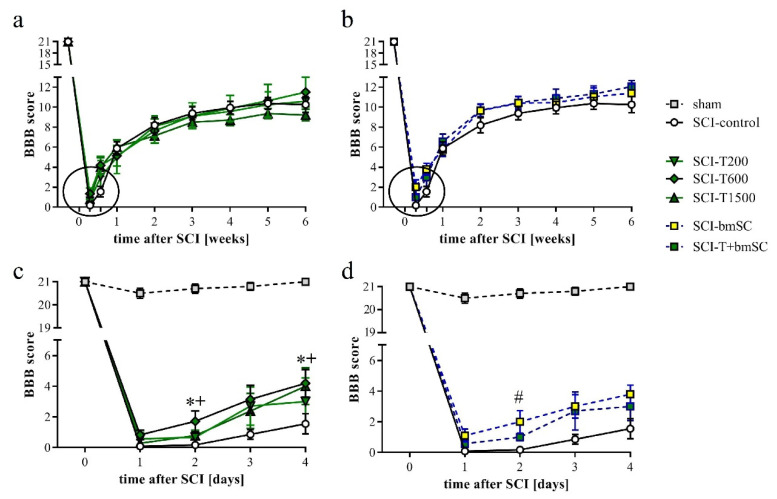
Recovery of motor functions after SCI assessed in the open field. Mean BBB scores were monitored before surgery, daily during the first 4 dpo after surgery, and then once per week during 6 weeks of evaluation. (**a**) Results for SCI-control, treated with saline, and the TUDCA treated groups. (**b**) Results for SCI-control and the SCI groups treated with bmSC and TUDCA + bmSC. (**c**,**d**) Changes in the acute/subacute phase; effects of treatment with 2 × 300 mg/kg TUDCA or with bmSC were significant at 2 dpo and/or 4 dpo [see main text for statistical evaluation; * (SCI-T600), + (SCI-T1500), # (SCT-bmSC) indicate *p* < 0.05; error bars indicate SEM]. Recovery of motor function improved in all treatment groups until a plateau was reached after three weeks; experimental groups are indicated as for Figure 2.

One day after SCI, the rats’ ability to use their hind legs was assessed in the open field. The SCI caused paralysis, as indicated by no or only slight movement of joints. Laminectomy without contusion resulted in temporary gait instability in some cases. Two TUDCA-treated SCI animals, which presented a BBB score above 2 at 1 dpo, were excluded from the analysis because we considered this an indication of a less severe SCI rather than an effect of treatment (indicated in Figure 1a). With time after injury, sensory-motor functions of the rats improved significantly, and different treatments were effective [two-factor ANOVA, time after SCI: F (9, 470) = 121, *p* < 0.0001 (pre-SCI data not included); treatment effect: F (5, 470) = 3.1, *p* < 0.01 (laminectomy group not included); interaction: F (45, 470) = 0.7, n.s.]. During the first 4 dpo, the majority of treated animals recovered the ability to move their hind legs. After treatment with 300 mg/kg TUDCA, this recovery occurred faster, such that BBB scores were significantly higher at 2 and 4 dpo compared to saline treatment (Figure 3a,b, subacute phase of these groups expanded in c,d). Improvements after two injections of 100 mg/kg TUDCA were not distinguishable from results after saline injections. Recovery of motor function improved further between one and three weeks after lesion, when all rats showed plantar stepping and weight support at least in stance but usually no coordination between fore and hind limbs (BBB 9–11). No improvement beyond this level was observed in the chronic phase (Figure 3a,b). Combinatorial therapy with TUDCA and bmSC or bmSC alone achieved higher BBB scores at 1–6 weeks (ΔBBB 0.7–1.9 higher than SCI-control, ΔBBB 0.5–1.2 higher than SCI-T200; *p* < 0.05), but there were no significant differences between treatment with bmSC and combinatorial treatment. The experiment confirmed previous results with bmSC injection only [8].

**Figure 4:**
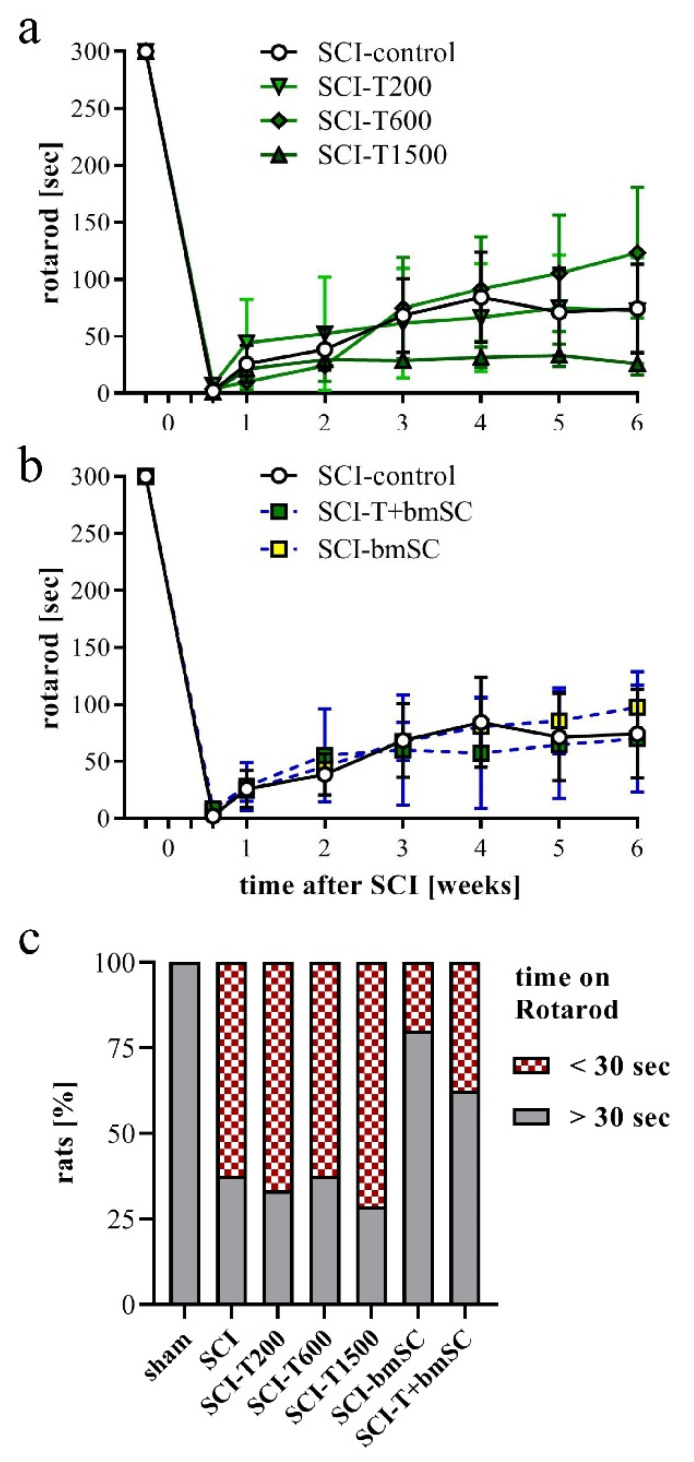
Recovery of motor functions after SCI assessed with the Rotarod assay. At 4 dpo, we confirmed that no SCI treated animals showed weight supported steps. Beginning at 7 dpo, some rats in all groups showed gradual improvement. (**a**,**b**) Time that rats were able to keep their balance on the rotating bar; mean +/− SEM, no significant differences between groups were detected. (**c**) Percentage of rats that performed the Rotarod task for more than 30 s at 6 W after SCI (n = 7–8 rats/group). Experimental groups are indicated as in Figure 2.

In addition to the assessment in the open field, rats were subjected to the Rotarod test, which measures their ability to maintain equilibrium on a rotating bar. Before SCI, all rats had been trained to perform this task for at least 300 s, and at 4 dpo, none of the animals that met the BBB inclusion criterion was able to do so. Spontaneous recovery caused a significant increase in Rotarod score during the first four weeks in all animals (Figure 4a,b). No further improvement occurred subsequently [ANOVA, effect of time after SCI: F (3, 352) = 22.0, *p* < 0.001; treatment effect: F (5, 352) = 1.5, n.s., interaction: F (15, 352) = 0.8, n.s.]. Some animals which showed weight supported plantar steps in the open field refused to hold on to the bar. In the absence of an independent criterion to distinguish between voluntary refusal and inability to perform the task, no data were excluded from the evaluation, resulting in a high variability in this assay. An additional assessment using the percentage of rats that at 6 W maintained themselves for longer than 30 s on the Rotarod showed no improvement at all following TUDCA treatment but revealed that more of the rats in the groups treated with bmSC or TUDCA + bmSC were able to do this (Figure 4c). With eight animals per group, the statistical power did not suffice for this effect to be significant.

### 3.6. Effect of TUDCA Treatment on Neuroinflammation

**Figure 5:**
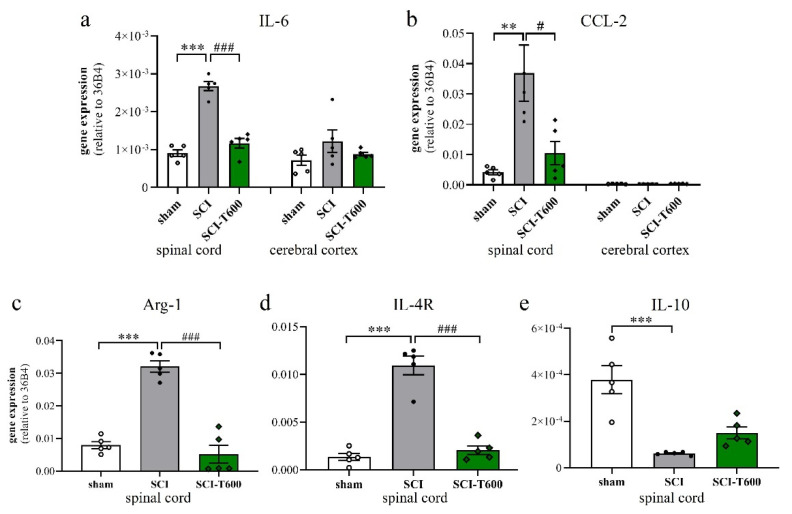
Expression of marker genes of inflammation. At 4 dpo, RNA extracts from spinal cord and cerebral cortex were analyzed with quantitative RT-PCR (treatment conditions abbreviated as in Figure 2). (**a**) Gene expression of IL-6 [ANOVA, spinal cord: F (2, 12) = 71.6, *p* < 0.0001; cortex: n.s.]; (**b**) CCL-2 [ANOVA, spinal cord: F (2, 12) = 8.9, *p* < 0.01; cortex: n.s.]; (**c**) arginase-1 [ANOVA, F (2, 12) = 56.0, *p* < 0.0001], (**d**) IL-4Rα [ANOVA, F (2, 12) = 66.0, *p* < 0.0001], (**e**) IL-10 [ANOVA, F (2, 12) = 18.7, *p* < 0.001]. Significant differences detected with post hoc Dunnett’s tests (*p* < 0.05, *p* < 0.001, *p* < 0.001) are indicated with **, *** symbols for SCI vs. laminectomy and #, ### for SCI vs. SCI-T600.

To a large degree, the devastating effects of SCI are due to the neuroinflammatory response of microglia and blood-derived macrophages. Since TUDCA is known for its anti-inflammatory effect on microglia, we confirmed this for our study by measuring the expression of marker genes in the spinal cord at 4 dpo. This experiment was done for the high dose of TUDCA. As in previous experiments, SCI caused mRNA upregulation of the inflammatory cytokine IL-6 and chemokine CCL-2 in spinal cord extracts. This appeared to be a local response and was not found in the cerebral cortex (Figure 5a,b). Treatment with TUDCA significantly reduced the expression of these and other (Table S1) inflammatory genes in the spinal cord. The lesion also caused alternative activation of microglia/macrophages, which was demonstrated by a highly significant increase in transcription of arginase-1 and IL-4R (Figure 5c,d). Contrary to our expectation, the bile acid treatment also reduced this response, indicating that it did not promote differentiation of an M2 phenotype. The cytokine IL-10 promotes alternative activation of macrophages and is produced by a subpopulation of these cells. At 4 dpo, we found a significant reduction of IL-10 transcripts in the spinal cord. This also occurred after TUDCA treatment but, compared to the SCI-controls, was no longer significant (Figure 5e).

**Figure 6:**
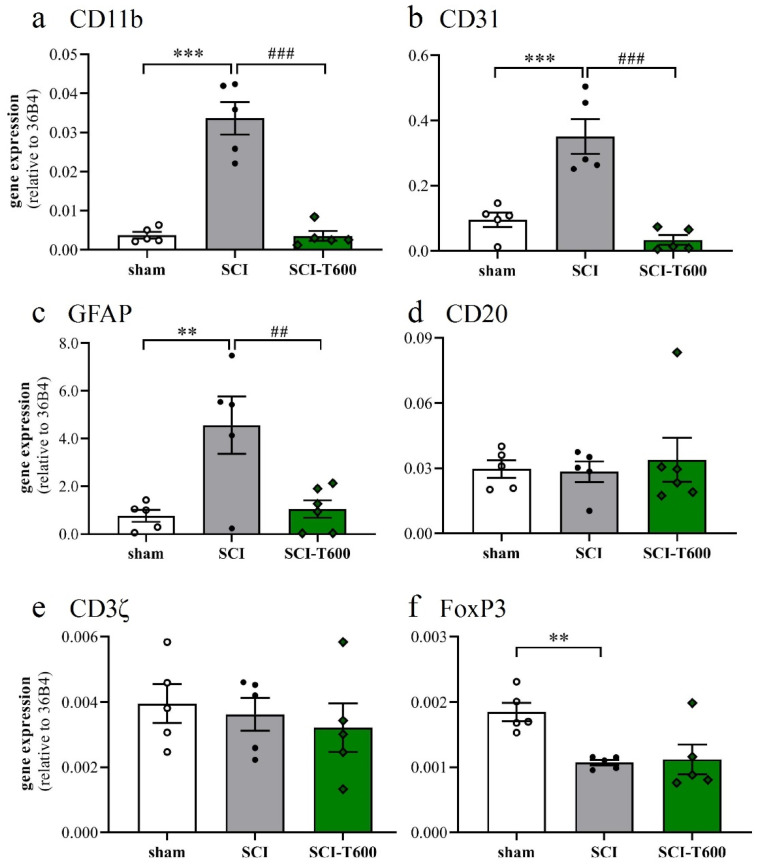
Expression of marker genes of cell type activation. At 4 dpo, RNA extracts from the spinal cord and cerebral cortex were analyzed with quantitative RT-PCR (treatment conditions abbreviated as in Figure 2). (**a**) Gene expression of CD11b, marker of microglia and macrophage [ANOVA, F (2, 12) = 46.3, *p* < 0.0001]; (**b**) CD31, endothelial cells, macrophages and lymphocytes [ANOVA, F (2, 12) = 23.9, *p* < 0.0001]; (**c**) GFAP, astrocytes [ANOVA, F (2, 12) = 8.6, *p* < 0.01]; (**d**) CD20, B-cells [ANOVA, F (2, 12) = 1.6, n.s.]; (**e**) CD3ζ, T-cells [ANOVA, F (2, 12) = 2.2, n.s.]; (**f**) FoxP3, regulatory T-cells [ANOVA, F (2, 12) = 7.7, *p* < 0.01]. Significant differences detected with post hoc Dunnett’s tests (*p* < 0.05, *p* < 0.001, *p* < 0.001) are indicated with **, *** symbols for SCI vs. laminectomy and ##, ### for SCI vs. SCI-T600.

The inflammatory activation of microglia or macrophages was also reflected by the expression of the complement receptor 3A (integrin αM, CD11b), which increased 7-fold after SCI. Similar to the other pro-inflammatory markers, its expression was significantly reduced after treatment with TUDCA (Figure 6a). Platelet endothelial cell adhesion molecule-1 (CD31) is found on endothelial cells, macrophages, and various lymphocytes. Its expression was detected in the control tissue, increased after SCI, and also was much reduced after TUDCA treatment (Figure 6b). Activation of astrocytes in neuropathologies is associated by an upregulation of the glial fibrillary acidic protein (GFAP). Transcripts of this gene, which was highly expressed in the spinal cord, increased 4-fold after SCI, and the response was inhibited by bile acid injections (Figure 6c). The validity of these effects on gene regulation can be appreciated by comparison with marker genes of other cell types, such as B lymphocytes (CD20; Figure 6d) and T lymphocytes (CD3ζ; Figure 6e). Indicators of these cells did not significantly change at 4 dpo with or without TUDCA injections. A marker gene of regulatory T-cells (FoxP3; Figure 6f) was expressed at a lower level after SCI and not influenced by treatment.

**Figure 7:**
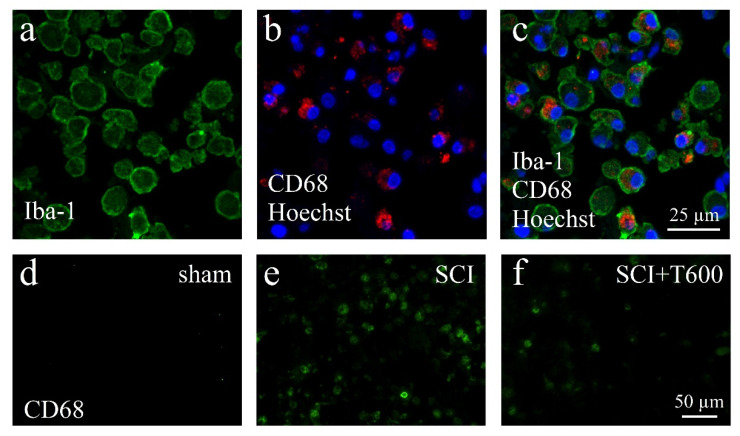
Activation of macrophages after SCI. (**a**–**c**) Confocal microscopy images show examples of macrophages in the SCI lesion center at 4 dpo. All cells are IR for Iba-1 (**a**, green) and most also for CD68 (**b**, red), shown in combination with nuclear staining (**b**,**c**, Hoechst 33342, blue). (**d**–**f**) CD68 IR macrophages in the spinal cord after laminectomy (**d**, sham), SCI (**e**), and SCI 2 × 300 mg/kg TUDCA treatment (**f**). Same magnifications are used in (**a**–**c**) and in (**d**–**f**) (scale bars in (**c**,**f**)).

**Figure 8:**
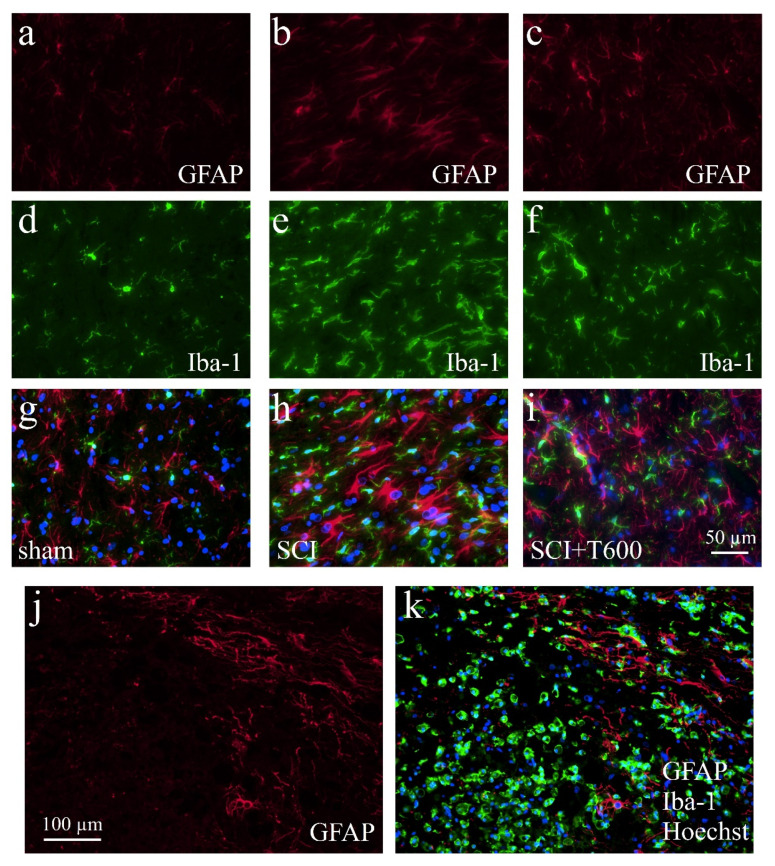
Activation of microglia and astrocytes after SCI. The IF photographs show examples of grey matter at 4 dpo and a distance of ca. 0.4 mm from the lesion center, which were double-stained for astrocytes (GFAP; **a**–**c**) and microglia (Iba-1; **d**–**f**). Photographs were superimposed with additional nuclear staining (Hoechst 33342; **g**–**i**). (**a**,**d**,**g**) Section from laminectomy-operated animal. (**b**,**e**,**h**) SCI with saline injection; (**c**,**f**,**i**) SCI treated with two injections of TUDCA 300 mg/kg. (**j**,**k**) At 4 dpo under all SCI treatment conditions, astrocytes showed increased GFAP IR but a glial scar had not yet developed. Scale = 50 μm in (**i**) (for (**a**–**i**), 40× objective) and 100 μm in (**j**) (for (**j**,**k**), 20× objective). Macrophages fill the lesion center.

The lesion-induced activation of microglia and astrocytes was confirmed by microscopical inspection using double-staining IF for Iba-1, CD68, and GFAP (Figure 7 and Figure 8). As expected from previous experiments, SCI disrupted the blood spinal cord barrier, caused an influx of hematogenous macrophages, and activated resident microglia and astrocytes. At 4 dpo and 6 weeks, under all treatment conditions, the center of the lesion was filled with cellular debris and Iba-1/CD68 positive cells. These made up 8.3 ± 1.1% of the cells in the center of the lesion (ROI 0.075 mm^2^; Figure 7a–c). The majority of CD68 positive cells were likely to be of hematogenous origin, and these were absent in the spinal cords of animals that had received only the laminectomy. Already at 4 dpo, the lesion center was devoid of neurons (NeuN IR) and astrocytes (GFAP IR). After TUDCA treatment, 5.7 ± 1.0% of the cells in this area were macrophages, as identified by morphology and CD68 IR. With increasing distance from the lesion center, the number of CD68 positive cells decreased such that at 8 mm posterior and anterior distances, only a few macrophages were observed in the SCI animals. Therefore, distance to the lesion significantly affected the strength of the CD68 signal, and a treatment effect was only significant at the central location (see quantification below).

### 3.7. Effect of TUDCA Treatment on Glial Activation after SCI

**Figure 9:**
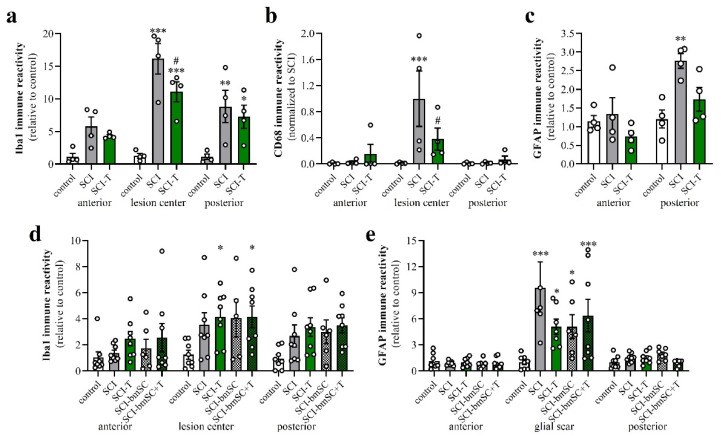
Activation of macrophages, microglia and astrocytes after SCI. Data from spinal cord areas were normalized for IR in the anterior position of sham-operated animals and analyzed with 2-factor ANOVA followed by post hoc Sidak tests. (**a**) Quantification of Iba-1 IR (fluorescence integrated density) at 4 dpo in white matter at 8 mm anterior, 8 mm posterior of the lesion site and in the lesion center [2-factor ANOVA, location effect: F (2, 27) = 11.7, *p* < 0.01; treatment effect: F (2, 27) = 29.9, *p* < 0.001; interaction: F (4, 27) = 3.2, *p* < 0.05]. (**b**) Quantification of CD68 IR at 4 dpo in areas 8 mm anterior and posterior of the lesion site and in the lesion center [2-factor ANOVA, location effect: F (2, 27) = 6.7, *p* < 0.01; treatment effect: F (2, 27) = 3.3, *p* = 0.05; interaction: *p* < 0.05; data were normalized to SCI, lesion center, as almost no CD68 cells are found outside this area]. (**c**) Quantification of GFAP at 4 dpo in white matter at 8 mm anterior, 8 mm posterior of the lesion site [location effect: F (1, 18) = 14.2, *p* < 0.01; treatment effect: F (2, 18) = 6.6, *p* < 0.01; interaction: F (2, 18) = 3.4, n.s.]. (**d**) Quantification of Iba-1 IR at 6 W in white matter 8 mm anterior, posterior and in the lesion center site [location effect: F (2, 98) = 5.9, *p* < 0.01; treatment effect: F (4, 98) = 5.4, *p* < 0.001; interaction: F (8, 98) = 0.3, n.s.]. (**e**) Quantification of GFAP IR at 6 W in white matter 8 mm anterior and posterior of the lesion center and in the glial scar [location effect: F (2, 97) = 36.1, *p* < 0.001; treatment effect: F (4, 97) = 4.1, *p* < 0.01; interaction: F (8, 97) = 2.5, *p* < 0.001]. Significant differences detected with post hoc comparisons tests (*p* < 0.05, *p* < 0.001, *p* < 0.001) are indicated with *, **, *** for SCI vs. control and # SCI vs. SCI-T600].

Increasing the IR of GFAP (Figure 8a–c,g–i) and Iba-1 (Figure 8d–i) indicated activation of astrocytes and microglia at 4 dpo. In the anterior and posterior of the SCI site, both signals were stronger than in animals that had only received the laminectomy. Contusion injury caused a 15-fold increase in Iba-1 IR in and close to the lesion center compared to levels in sham-operated animals. After treatment with 2 × 300 mg/kg TUDCA, Iba-1 and CD68 signals were significantly reduced in the lesion center (Figure 9a,b). Morphological changes of GFAP positive cells around the lesion site indicated the activation of astrocytes at 4 dpo (Figure 8j,k). Quantification of the GFAP IR confirmed this observation for the white matter posterior of the lesion center. Astrocyte activation was less pronounced (i.e., not significantly different from laminectomy treatment) after bile acid treatment (Figure 9c).

At 6 W after SCI, Iba-1 IR was no longer as elevated as at 4 dpo but still 3- to 4-fold higher than in non-lesioned tissue. No significant differences between TUDCA-, bmSC- or TUDCA/bmSC-treated and saline-treated animals were observed in the chronic phase (Figure 9d). At this time, a prominent glial scar had formed in all SCI animals. Quantification of GFAP IR in this area (Figure 9e) confirmed a strong increase from the acute to the chronic phase after SCI (note different scales of *y*-axis in panels Figure 9c,e). The scar area appeared to be reduced by bile acid/bmSC treatment, but the differences in GFAP intensity did not reach significance.

**Figure 10:**
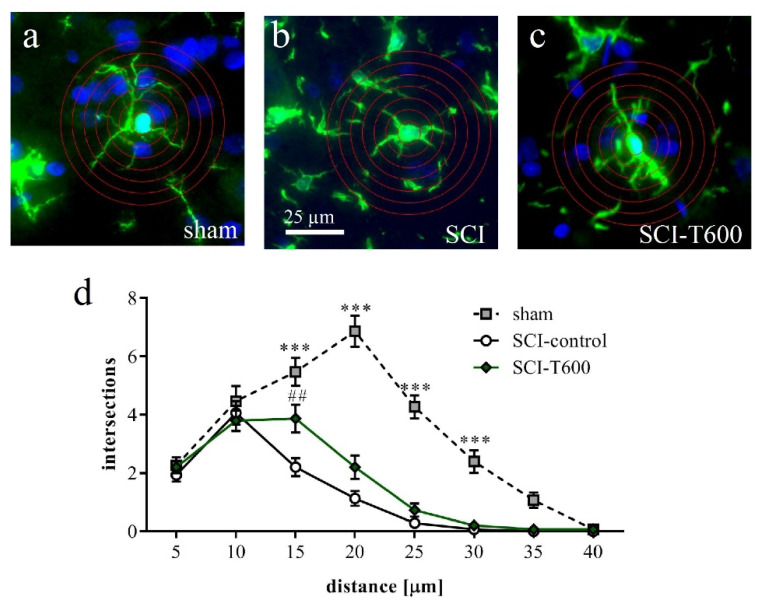
Effect of SCI and TUDCA treatment on microglia morphology. (**a**–**c**) Examples of Iba-1 IR cells in the spinal cord gray matter at 8 mm distance from the injury site at 4 days after sham operation (**a**), SCI control (**b**) and SCI with TUDCA treatment (**c**) are shown with superimposed rings for Sholl analysis. (**d**) Number of intersections of cellular processes at 5 µm intervals from the cell soma [two-factor ANOVA, distance from soma F (7, 335) = 78.6, *p* < 0.0001; treatment effect: F (2, 335) = 107, *p* < 0.001; interaction: F (14, 335) = 12.9, *p* < 0.001, results of Bonferroni multiple comparison tests are indicated with *** (*p* < 0.001) for sham vs. SCI-control and sham vs. SCI-T600 and with ## (*p* < 0.01) for SCI vs. SCI-T600]. All panels have the same magnification (scale bar in **b**).

Responding to an inflammatory environment, microglia cells are known to respond with a morphological transformation from a branched appearance to an ameboid shape. We confirmed this using a Sholl analysis of Iba-1 IR cells in gray matter at an 8 mm distance from the lesion center (Figure 10a–c). The morphological change associated with SCI was highly significant. Microglia cells in SCI injured animals at 4 dpo had fewer branches than in rats with laminectomy. This change was less pronounced after TUDCA treatment (*p* < 0.001), corroborating the hypothesis that the bile acid affected microglial activation.

### 3.8. Effect of TUDCA Treatment on SCI-Induced Apoptosis

Several groups reported that TUDCA treatment reduced cell death after SCI [22,24,25,26]. We found previously that the bmSC preparation used here was also cytoprotective [8]. To allow comparison with these publications, we evaluated cellular apoptosis in the lesion center and at 4 mm anterior and posterior to this position (Figure S1). Four days after SCI, we found 11.3 ± 3.1% (mean ± SD) of the cell nuclei in the lesion area to be TUNEL positive, whereas sham operated rats had only 0.40 ± 0.35% apoptosis in the white matter at the respective position (*p* < 0.001). Treatment with two injections of 300 mg/kg TUDCA significantly reduced TUNEL staining in the lesion area to 5.9 ± 4.2% (*p* < 0.05). In the ventral white matter at positions anterior and posterior of the lesion center, we found a slight, non-significant increase in the number of apoptotic cells at 4 dpo (control: 0.2 ± 0.3%, SCI: 1.1 ± 0.7%), which was not affected by treatment (SCI-T600: 1.1 ± 0.3%). The analysis of TUNEL staining in the chronic phase after SCI revealed continuing high levels of apoptosis without (SCI: 7.6 ± 18.6%) and with TUDCA treatment (SCI-T600: 16.4 ± 17.1%), indicating that bile acid injections had no lasting effect on cell survival (*p* > 0.1). In rats treated with bmSC, the proportion of apoptotic cells at 6 W was lower (SCI-bmSC: 4.1 ± 7.8%, *p* < 0.05), but there was no significant effect due to additional treatment with TUDCA (SCI-T + bmSC: 1.4 ± 1.0%, *p* < 0.05 vs. SCI; *p* > 0.1 vs. SCI-bmSC).

## 4. Discussion

In the present study, we found short term but no lasting benefits of TUDCA treatment on the recovery of bladder control and motor function after SCI in rats. Transcripts of the bile acid receptor TGR5 were detected in the spinal cord at a similar level of expression as in the brain. When combined with bmSC implants, TUDCA applications in the acute phase did not provide additional therapeutic benefit. While we confirmed previously reported anti-inflammatory and cytoprotective effects of TUDCA, our data indicate that the bile acid also reduced expression of genes that are associated with the M2 phenotype. The spinal cord contusion injury caused a transient loss of body weight in the rats, which was not significantly affected by TUDCA- or bmSC-treatment.

### 4.1. Are Bile Acids Promising for the Treatment of SCI?

There is good evidence that bile acids, specifically TUDCA and the TGR5 ligand INT777, modulate the activity of macrophages and microglia. Activation of TGR5 inhibits NFκB signaling and subsequent expression of inflammatory genes [29,30]. In LPS-treated microglia cells, bile acid treatment reduces nitrite production, expression of pyruvate kinase M2, which can act as a transcription factor, and downstream genes, such as lactate dehydrogenase [18]. Anti-inflammatory treatment is currently the only pharmacological approach for SCI in patients.

So far, five studies have been published to test TUDCA in rat models of SCI. The scientists used a weight drop device that caused a contusion similar to the present experiments [22,23,24,26] or a compression model, also at vertebral level T9, which was less severe and only damaged the dorsal columns [31]. In these studies, motor recovery was monitored during the subacute phase (5 dpo: [22,23,26]; 7 dpo: [31]; 10 dpo: [24]). To the extent that the data are comparable, i.e., severe T9 contusion injury and evaluation using the BBB scale, our present data confirm the reported outcomes. In addition, we found that TUDCA-treated rats recovered the autonomic control of bladder function earlier than under control conditions or after bmSC injection. However, our main conclusion is that TUDCA treatment does not provide lasting benefits after SCI: Extending the previous studies, we investigated effects in the chronic phase. In all rats, the improvement of motor function reached a limit after 3–4 weeks, which did not significantly differ between treatment groups except for the effect of additional bmSC treatment. High doses of TUDCA (5 × 300 mg/kg) were not more effective than lower ones, an observation that was also made with mice, where one injection of 100 mg/kg TUDCA gave better results than higher doses [25]. Another mouse study has recently been published with even higher doses, using fourteen administrations of 200 mg/kg TUDCA per os [32]. Since the longest treatment regime that we used terminated at 5 dpo, we do not rule out the possibility that continued application would extend the cytoprotective effects into the chronic phase and have therapeutic benefits.

The results with mice are not directly comparable with our data as the histological evaluation at 14 dpo demonstrated only minor effects of the SCI while motor scores were lower (BBB = 5, control SCI) than in our experiments (BBB = 8.2, control SCI; the rat scale was used in the mouse study as well). Behavioral evaluation was performed for up to 14 days, when TUDCA-treated mice reached a motor score of 10. We do not know whether recovery had reached a plateau or would have improved further in the following weeks. It is certainly possible that the continued application of TUDCA extended the beneficial effect into the chronic phase. Hou and colleagues also performed additional experiments regarding the therapeutic mechanism. These showed that TUDCA was not only neuroprotective but also improved axonal growth [32].

Most available data obtained with cell cultures indicates that TUDCA reduces inflammation, production of reactive oxygen species and ER stress by binding TGR5, subsequent cAMP synthesis and PKA activation [13,30,32]. Our rationale for the combinatorial therapy of TUDCA with bmSC was that the moderating influence on macrophages may improve survival of the stem cells. In a recent experiment we found a beneficial effect of bmSC treatment compared to methylprednisolone but were not able to detect the implanted cells later in the tissue [8]. The present experiments revealed no additional benefit of TUDCA with stem cells compared to stem cells alone. While the inhibitory effect of TUDCA on the release of inflammatory mediators was confirmed, there are more potent anti-inflammatory drugs available, which also do not provide a satisfactory therapy of SCI [2,33]. Furthermore, we found that TUDCA treatment rather decreased the alternative monocyte activation in the tissue. It is questionable whether the transient improvement of motor recovery and bladder function observed with TUDCA is meaningful from a clinical point of view.

Since application of 5 × 300 mg/kg TUDCA was associated with more weight gain, but 2 × 100 mg/kg and 2 × 300 mg/kg with less weight gain than observed in SCI-controls (post hoc tests n.s.), this parameter does not seem to be affected by treatment. Animals injected with bmSC showed a similar rate of recovery as the controls. In a previous study using the same type of bmSC, a stronger weight gain was observed with this treatment [8]. In those experiments, the bmSC-treated rats also suffered a more severe weight loss during the first days after SCI, which may have contributed to the relative increase in body weight thereafter. This was not the case in the present study.

### 4.2. How Does TUDCA Influence the Recovery of Urinary Function?

Autonomic dysfunctions, which have a large impact on the quality of life of SCI patients, include impairments of bladder storage and emptying. Contraction of the bladder is mediated by parasympathetic efferents from the sacral spinal cord via the pelvic nerve to the detrusor muscle of the bladder. Relaxation of the external urethral sphincter is controlled by somatic innervation from sacral segments via the pudendal nerve. These neural circuits are controlled by a coordination center in the reticular formation in the pons and midbrain [34]. Its activity is required for the voiding reflex [35]. The thoracic SCI employed in the present study disconnects the pontine from the sacral micturition centers, and this may cause the rats’ inability to urinate spontaneously [36]. Additional sympathetic innervation of the urethra originates in the lumbar segments of the spinal cord. These are also posterior to the SCI and therefore separated from the supraspinal areas. While in human SCI patients, neurogenic bladder dysfunction is generally irreversible [37], all rats recovered their ability to urinate spontaneously. This, in our experience, is generally observed even after the severe contusion injury (2 N) performed with the *Infinite Horizon* impactor. In clinical terms, the transient depression of spinal reflexes caudal to a SCI has been defined as “spinal shock”. Why these reflexes return is not completely understood [38].

Severed fibers in the spinal cord that connect with supraspinal centers are unlikely to be restored during the first two weeks after surgery, when the rats recover the ability to urinate spontaneously. Therefore, the most probable explanation for the functional regeneration is that spared fibers in ventral white matter tracts are dysfunctional during the subacute phase after SCI and recover from spinal shock in the subsequent days. Treatment with TUDCA may accelerate tissue remodeling, e.g., by increasing the release of TGFβ [39]. It is also conceivable that plastic changes in the sacral micturition centers occur after the descending innervation is lost. As some neurons express TGR5 [12,40], it is an intriguing possibility that bile acids may affect neuronal plasticity. To our knowledge, this has not been investigated so far. Finally, the observed benefit may have been a non-specific side effect of the systemic anti-inflammatory activity of the TUDCA injections.

### 4.3. How Does TUDCA Affect the Inflammatory Phenotype after SCI?

Our data on gene expression of CD11b, GFAP, IL-6, and CCL-2 confirm the inhibitory effect of TUDCA on inflammatory pathways. These qRT-PCR measurements at 4 dpo included a spinal cord segment of 2 cm including and surrounding the lesion site. Our histological evaluation of Iba-1 showed significantly lower IR near the lesion center, which is in accordance with the gene expression data. Evaluation of CD68 IR, absent in the non-injured tissue, also demonstrated an effect of bile acid treatment, indicating a lower number of inflammatory macrophages in the lesion site. At a distance of 8 mm, CD68 positive cells were observed in some animals only, without significant differences between groups. These data corroborate previous results where TUDCA reduced Iba-1 IR in the hippocampus of LPS-treated mice [17].

In the context of neuro-inflammation, two phenotypes of macrophages and activated microglia are frequently distinguished, referred to as M1 (pro-inflammatory) and M2 (anti-inflammatory or alternative activation); [41]. The M2 state has been subdivided further to accommodate differential patterns of gene expression. Experimentally, the M1 phenotype may be defined as the outcome of stimulation with TNFα and IFNγ, the M2a phenotype as the response to IL-4, and the M2c phenotype in response to IL-10 [42]. Based on the fact that cAMP induces M2 associated genes of microglia [43] and on histological data with a mouse model of systemic inflammation [17], we expected the TUDCA injections not only to reduce inflammation in the spinal cord [31], but also to stimulate the differentiation of the alternative phenotype of microglia. This was not the case. Rather, we observed at 4 dpo, a significant reduction of two classical M2 markers (arginase-1, IL-4Rα) and no effect on the third (IL-10). In cell culture experiments with microglia, bacterial lipopolysaccharides (LPS) increased IL-4α and IL-10 expression. TUDCA did not change this significantly and had no effect in the absence of LPS [17]. However, in the same study, in vivo injections of TUDCA induced arginase-1 IR and IL-10 mRNA (but not IL-4Rα) in the hippocampus of LPS-treated mice. Thus, the so-called “M2 phenotype” may not be a useful concept [44], and it appears as if TGR5 activation has different effects depending on other prevailing stimuli in the environment. At this point, we have no conclusive concept regarding the role of bile acids or even TGR5 in microglia differentiation.

Bile acids are also cytoprotective. This seems to involve signaling kinases of Akt and PI3K [40,45] and may be independent of the inhibition of NFκB and inflammatory signals. SCI studies with TUDCA showed a reduction of apoptosis [22,25,26,32], and our data with TUNEL staining confirms it. Again, the absence of lasting effects on cell survival and motor recovery suggests that the relevance of this for SCI is limited.

Despite this sobering assessment of our results, TGR5 may still be considered a target in neuropathologies. Prolonged treatment with bile acids [32] is an option to be explored. In rodents and primates, TUDCA is not a physiological, endogenous signal. It is conceivable that other bile acids and synthetic TGR5 ligands [46,47] reduce apoptosis more potently and elicit unknown benefits because cAMP, the second messenger of TGR5, is implicated in multiple pro-regenerative pathways. Inhibitory signals for axonal growth may be overcome by this mechanism [32]. Recently, oleanolic acid was successfully tested in a mouse model of SCI [48]. Bile acid applications are being investigated in other neuropathologies [49]. Apart from TGR5 signaling, bile acids activate a variety of nuclear receptors [15,50], and some of these are also promising targets in the context of SCI [51,52,53,54].

## 5. Conclusions

Treatment of SCI with the bile acid TUDCA reduces inflammation and improves recovery of autonomic and motor functions in the subacute phase. No lasting therapeutic benefits were observed in the chronic phase or in combinatorial treatment with bmSC. Therefore, our data do not support the use of TUDCA as a treatment of SCI, at least when this is done in the acute phase only. The efficacy of alternative agonists of the bile acid receptors TGR5 and FXR should be investigated.

## Figures and Tables

**Table 1:** Primer sequences used in quantitative RT-PCR.

Gene	Gene ID (NCBI Reference)	Primer Sequences	Product Tm [°C]	Product Size [bp]
*36B4*	AC130745.3	sense: TTCCCACTGGCTGAAAAGGT antisense: CGCAGCCGCAAATGC	60	60
*IL-6*	NM_012589	sense: TAGTCCTTCCTACCCCAATTTCC antisense: TTGGTCCTTAGCCACTCCTTC	60	76
*CCL-2*	NM_031530	sense: TGCTGTCTCAGCCAGATGCAGTTA antisense: TACAGCTTCTTTGGGACACCTGCT	64	131
*Arg-1*	NM_017134	sense: GCAGAGACCCAGAAGAATGGAAC antisense: CGGAGTGTTGATGTCAGTGTGAGC	62	144
*IL-4Rα*	NM_133380	sense: GATCTTCTGAGCCCGGTTGA antisense: CTCTCCGCTTGCTGCATT	59	59
*IL-10*	NM_012854	sense: GATGCCCCAGGCAGAGAA antisense: CCCAGGGAATTCAAATGCT	61	57
*CD11b*	NM_012711.1	sense: CTGCCTCAGGGATCCGTAAAG antisense: CCTCTGCCTCAGGAATGACATC	60	150
*CD31*	NM_031591	sense: GAGGTATCGAATGGGCAGAA antisense: GTGGAAGACCCGAGACTGAG	55	174
*GFAP*	NM_017009	sense: TGGCCACCAGTAACATGCAA antisense: CAGTTGGCGGCGATAGTCAT	60	134
*CD20*	NM_001107578	sense: TCTTGGGCATTCTGTCGGTG antisense: TCTACAACACCGGCTGTCAC	60	70
*CD3ζ*	NM_170789	sense: TCATACCCCAGCCCAGTTCT antisense: CGGCTCTGGGGACTTTACAA	60	148
*FoxP3*	NM_001108250	sense: TCATCACTGGCTTTCTGCGT antisense: GCTTTTAGCCTGAACCCCCT	60	95
*TGR5*	NM_177936	sense: AAAGGTGGCTACAAGTGCTTC antisense: TTCAAGTCCAAGTCAGTGCTG	58	103

See list for abbreviations, all gene sequences are from rat.

## Data Availability

Data supporting the reported results are available from the corresponding author upon reasonable request.

## Supplementary Materials

The following are available online at https://www.mdpi.com/article/10.3390/biomedicines10071501/s1, Table S1: Effect of SCI and TUDCA treatment on the expression of genes associated with microglial activation; Figure S1: SCI-induced apoptosis as shown with TUNEL staining.

## Abbreviations

36B4	acidic ribosomal phosphoprotein P0
Akt	serine/threonine protein kinase encoded by the oncogene in retrovirus isolated from stock A strain k mouse thymoma cell line (protein kinase B)
ANOVA	analysis of variance
BBB	Basso/Beattie/Bresnahan locomotor rating scale for the assessment of hind limb motor function in rats
bmSC	bone marrow-derived stromal cells
CCL	chemokine C-C motif ligand
CD	cluster of differentiation
DMSO	dimethyl sulfoxide
dpo	days post operation
EDTA	ehylenediaminetetraacetic acid
GFAP	glial fibrillary acidic protein
GMP	good manufacturing practice
Iba-1	ionized calcium-binding adapter molecule 1
IFN	interferon
IF	immunofluorescence
IgG	immunoglobulin G
IH	Infinite Horizon spinal cord impactor
IL	interleukin
i.p.	intraperitoneal
IR	immunoreactivity
LPS	lipopolysaccharide
NFκB	nuclear factor kappa B
PBS	phosphate buffered saline
PCR	polymerase chain reaction
PFA	paraformaldehyde
PI3K	phosphoinositide 3-kinase
PKA	serine-threonine protein kinase A family
PWT	paw withdrawal threshold
ROI	region of interest
RT	room temperature
s.c.	subcutaneous
SCI	spinal cord injury
SEM	standard error of the mean
T200, T600, T1500	treatment with TUDCA 2 × 100 mg/kg, 2 × 300 mg/k and 5 × 300 mg/kg respectively in the present study
TBS-T	Tris-buffered saline/Tween 20
TGF	transforming growth factor
TGR5	Takeda G protein-coupled receptor-5
TNF	tumor necrosis factor
TRITC	tetramethyl rhodamine iso-thiocyanate
TUDCA	tauroursodeoxycholic acid
TUNEL	terminal deoxynucleotidyl transferase dUTP nick end labeling
W	weeks after SCI
