# Supplementary material for: Tauroursodeoxycholic Acid Reduces Neuroinflammation but Does Not Support Long Term Functional Recovery of Rats with Spinal Cord Injury
Source: Biomedicines. 2022 Jun 25;10(7):1501. doi: 10.3390/biomedicines10071501 (PMC9313003; doi:10.3390/biomedicines10071501)
Supplement: Supplementary file 1 [file biomedicines-10-01501-s001.zip › biomedicines-1690432-supplementary.pdf]

Supplementary Materials

Table S1. Effect of SCI and TUDCA treatment on the expression of genes associated with microglial activation.

Gene	Expression; Mean, Normalized to 36B4			Dunnett's Post-Hoc Test	
	laminectomy	SCI, control	SCI-T600	Sham vs. SCI	SCI vs. SCI-T600
IL-6	1.00×10^{-3}	2.45×10^{-3}	1.03×10^{-3}	$p < 0.001$	$p < 0.001$
IL-12β	5.98×10^{-4}	3.33×10^{-4}	7.06×10^{-4}	n.s.	n.s.
CCL-2	0.44×10^{-2}	3.33×10^{-2}	1.84×10^{-2}	$p < 0.05$	n.s.
HMGB1	0.195×10^{-3}	0.449×10^{-3}	0.081×10^{-3}	n.s.	$p < 0.05$
IFNβ	4.99×10^{-4}	7.94×10^{-4}	4.66×10^{-4}	n.s.	$p < 0.05$
PKM2	0.285	0.687	0.178	$p < 0.05$	$p < 0.001$
arg-1	0.79×10^{-2}	3.21×10^{-2}	0.97×10^{-2}	$p < 0.01$	$p < 0.01$
IL-4R	0.20×10^{-2}	1.12×10^{-2}	0.30×10^{-2}	$p < 0.01$	$p < 0.01$
IL-10	3.49×10^{-4}	0.74×10^{-4}	1.91×10^{-4}	$p < 0.01$	n.s.

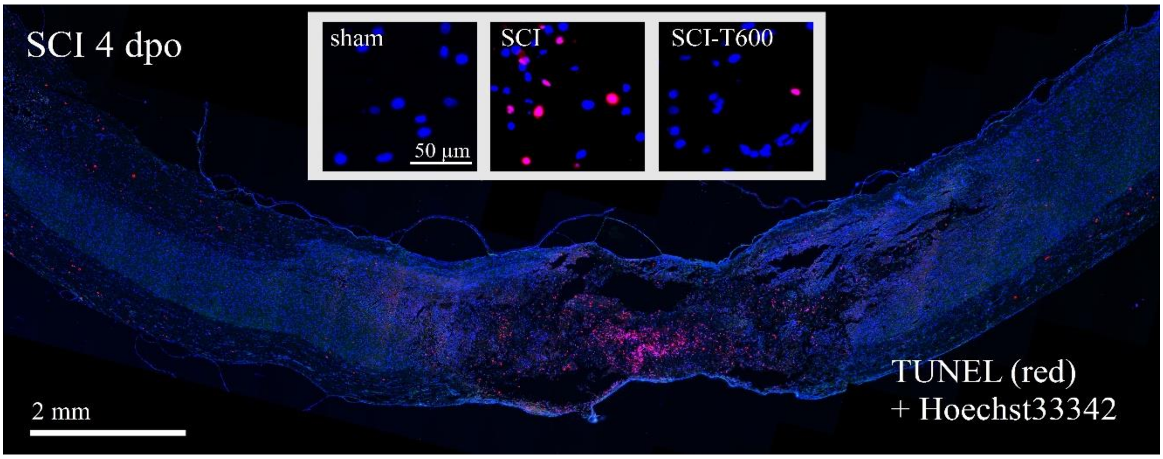

Figure S1. SCI-induced apoptosis as shown with TUNEL staining. Overview of a spinal cord section at 4 dpo with a high degree of apoptosis within the lesion center; magenta coloration indicates TUNEL fluorescence (red) of cell nuclei stained with Hoechst-33342 (blue). **Insets:** High magnification images of TUNEL staining at 4 mm distance from the lesion site after laminectomy (sham), SCI-control and SCI-T600.
